# Ethanol foam: a novel type of foam sclerosant for treating venous malformations

**DOI:** 10.3389/fneur.2024.1431723

**Published:** 2024-08-07

**Authors:** Han-Shu Zhang, Yi-Ran Liu, Shao-Hua Liu

**Affiliations:** ^1^Department of Oral and Maxillofacial Surgery, Qilu Hospital of Shandong University, Jinan, Shandong, China; ^2^Department of Endocrinology, Qilu Hospital of Shandong University, Cheeloo College of Medicine, Shandong University, Jinan, Shandong, China

**Keywords:** venous malformations, sclerotherapy, absolute ethanol, foam sclerosant, ethanol foam

## Abstract

**Introduction:**

Sclerotherapy is a commonly utilized treatment approach for venous malformations. Absolute ethanol is renowned for its remarkable efficacy as a potent sclerosants, but it is potentially associated with severe complications. Foam sclerotherapy is considered superior to liquid sclerotherapy owing to its heightened efficacy and diminished incidence of complications. Thus, our objective was to devise an ethanol foam sclerosant that delivers exceptional efficacy while mitigating complications.

**Methods:**

In the first set of experiments, we identified the suitable range of ethanol concentrations for sclerotherapy through human umbilical vein endothelial cell proliferation assays and blood clotting experiments. Next, the surfactants polysorbate 80, egg yolk lecithin, and hyaluronic acid were added to create stable ethanol foam, with their ratios meticulously optimized.

**Results:**

The optimal concentration range of ethanol was determined to be 30–60%. Eventually, a 48% ethanol foam was successfully produced with excellent stability. Other than ethanol, the formulation included 5 × 10^−3^ g/mL polysorbate 80, 10^−2^ g/mL egg yolk lecithin, and 0.04 mL/mL hyaluronic acid.

**Discussion:**

The novel ethanol foam produced here could be a promising candidate for the treatment of venous malformations.

## 1 Introduction

Venous malformations (VMs) are complex congenital lesions with diverse clinical presentations that can result in significant esthetic and functional limitations and even death in some cases (1). Currently, the primary treatment strategy for VMs is sclerotherapy, which involves the administration of sclerosants into the lumen of the vessel that consequently leads to venous wall damage and occlusion of the vessel (2). However, sclerosants are easily diluted and can be washed away into the bloodstream, and this limits sufficient contact with the inner walls of the lesion (3). Some commonly used sclerosants are absolute ethanol, polidocanol, and bleomycin (4–6), among which absolute ethanol is associated with the lowest incidence of lesion re-expansion (7, 8). However, absolute ethanol can cause severe complications (9) and, reportedly, has higher complication rates than other sclerosants (10). Improving the current sclerotherapy strategy and agents could help improve the treatment outcomes and quality of life of patients with VMs.

A potential alternative to the current sclerotherapy methods is foam sclerotherapy, a unique technology and has been found to improve the efficiency of sclerotherapy. Foam displaces blood from the lesion, thereby increasing the contact time between the sclerosant and vessel walls and improving the effect of the sclerosant (11). Another advantage of foam sclerotherapy is that it requires a lower dose of the agent. Based on the known advantages of foam sclerotherapy and absolute ethanol, we speculated that transforming ethanol from the liquid form to foam may be an ideal approach for sclerotherapy. When delivered in the form of a foam, the concentration of ethanol required will be lower, and this may reduce its associated complications while ensuring treatment efficacy. Accordingly, the aim of this study was to develop an ethanol foam sclerosant that can be used for the treatment of VMs.

## 2 Materials and methods

### 2.1 Cell proliferation experiments

We evaluated the effect of different concentrations of ethanol on the destruction of venous endothelial cells in a cell proliferation experiment using human umbilical vein endothelial cells (HUVECs; ScienCell, San Diego, California, United States).

The experiment included one blank group (no cells), one control group in which cells were cultured in endothelial cell medium (ECM, ScienCell), one control group that was treated with absolute ethanol, and nine experimental groups (treated with ethanol concentrations of 10%, 20%, 30%, 40%, 50%, 60%, 70%, 80%, and 90% in volumetric fractions). First, HUVECs were seeded in 96-well plates at a density of 2.5 × 10^3^ cells/well and cultivated in ECM for 24 h. To evaluate the transient effect of ethanol on HUVECs, ECM was immediately added to the wells after the addition of the experimental solution to dilute ethanol, which was then removed via suction. The plate was washed three times with ECM for 3 min. Next, 50 μL of ECM and 20 μL of Cell Counting Kit-8 reagent (CCK-8; Beyotime, Shanghai City, China) were added, and the plates were placed in an incubator at 37°C with a 5% CO_2_ atmosphere for 1 h. The cell viability of HUVECs was determined based on absorbance of different samples at a wavelength of 550 nm using a spectrophotometer (UV-VIS, UV1800; Shimadzu, Japan).

### 2.2 Blood clotting experiments

To determine the blood clotting ability of ethanol at different concentrations, fresh coagulated blood from a healthy volunteer (1 mL each tube) was added to 11 centrifuge tubes comprising two control tubes (containing distilled water or absolute ethanol) and nine experimental tubes (containing ethanol at the same concentrations as those used in the cell proliferation experiments). After the sample solutions were mixed with blood, we observed changes in the color and state of the blood to determine the blood clotting ability of ethanol.

### 2.3 Foam production

Tessari’s method was used to produce foam with a gas–liquid ratio of 3:1. We tried to determine whether all the liquid could be converted to foam. Furthermore, the foam half-life time (FHT), defined as the time required for one volume of foam to be reduced to half its original volume, was used to assess foam stability.

We used polysorbate 80 (Tween 80) (injection quantity, 500 g; Nanjing Weier Pharmaceutical Co. Ltd., Jiangsu, China) and egg yolk lecithin (PC 80) (injection quantity, 50 g; Shenyang Tianfeng Biopharmaceuticals Co. Ltd., Liaoning, China) as surfactants to produce ethanol foam in experiments A and B, respectively. In experiment A, Tween 80 was used at concentrations of 1.6 × 10^−5^, 10^−4^ g/mL, 10^−3^, 5 × 10^−3^ g/mL, and 10^−2^ g/mL. The fractional volumes of ethanol used were 40, 42, 44, 46, 48, 50, 52, 54, 56, 58, and 60%. In experiment B, the concentrations of PC 80 were 10^−2^ g/mL, 1.5 × 10^−2^ g/mL, and 2 × 10^−2^ g/mL. Next, the combined effect of PC 80 and Tween 80 was evaluated in experiment C. For this, we added 5 × 10^−3^ g/mL Tween 80 and 10^−2^ g/mL PC 80 to the ethanol solution based on the results of experiments A and B. The fractional volume of ethanol used was found to be the same in experiments A, B, and C. Finally, to improve the stability of the ethanol foam, we performed experiment D, in which hyaluronic acid (HA) (20 mg/2 mL, Sofast sodium hyaluronate injection; Shandong Bauschfruida Pharmaceutical Co. Ltd., Shandong, China) was used along with the same concentrations of PC 80 and Tween 80 as experiment C, and the fractional volume of ethanol used was 48%. We added HA at incremental concentrations of 0.01 mL/mL ranging from 0 to 0.08 mL/mL.

## 3 Results

### 3.1 Effect of ethanol on cell proliferation ability

**Figure 1:**
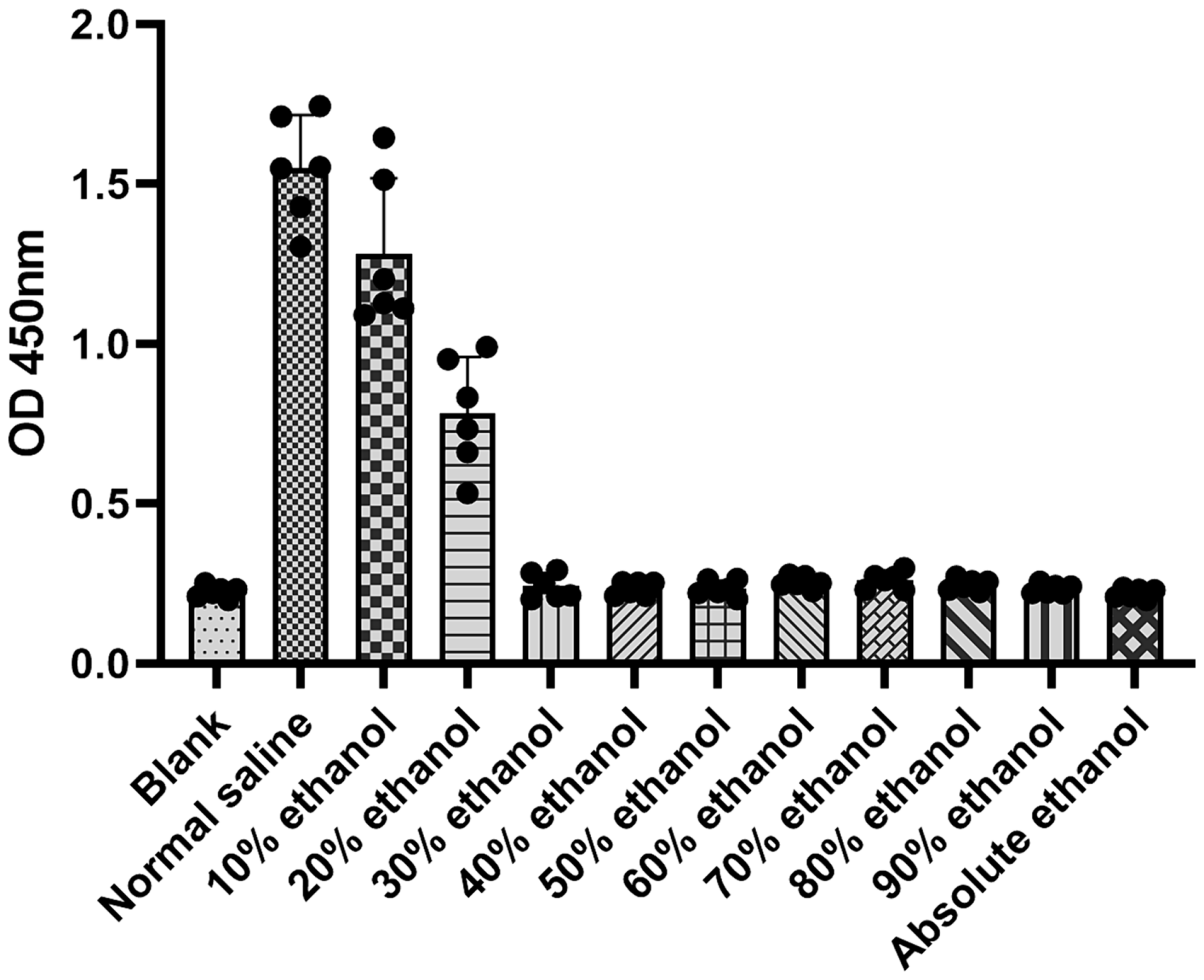
Proliferation assay of HUVECs incubated with different concentrations of ethanol.

In the HUVEC proliferation assay, the destructive effect of ethanol increased with increasing concentrations of ethanol. When the cells were treated with ethanol at concentrations above 30%, the absorbance was not significantly different from that of the blank and absolute ethanol groups (Figure 1). This indicates that all the cells were destroyed at ethanol concentrations above 30%.

### 3.2 Blood clotting ability of ethanol

**Figure 2:**
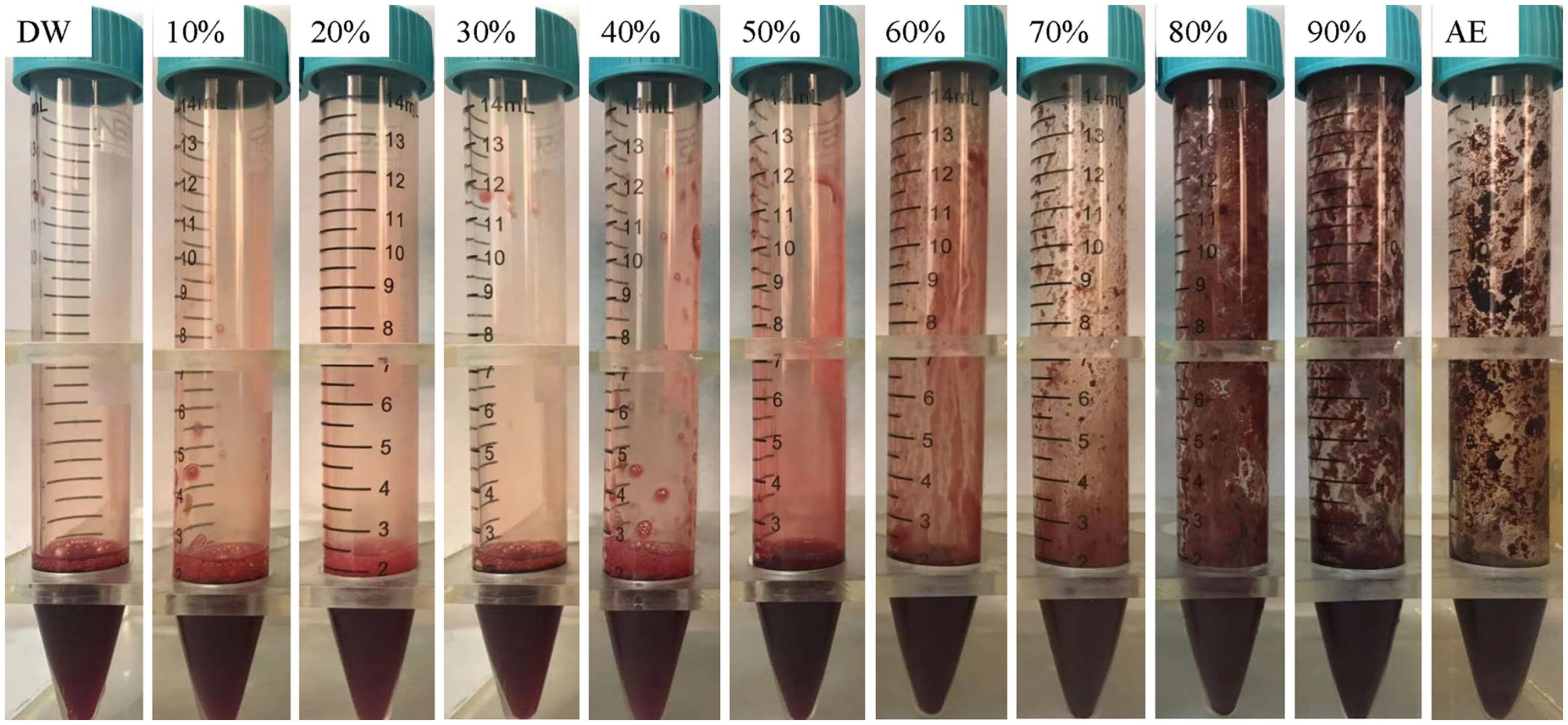
Blood clotting ability of ethanol at different concentrations. DW, distilled water; AE, absolute ethanol.

The blood clotting ability was found to increase with increasing ethanol concentrations (Figure 2). When the blood was mixed with ethanol at concentrations below 60%, there was no obvious visible clotted blood. This implies that ethanol can induce clotted blood only at concentrations above 60%.

### 3.3 Efficiency of ethanol foam production

**Figure 3:**
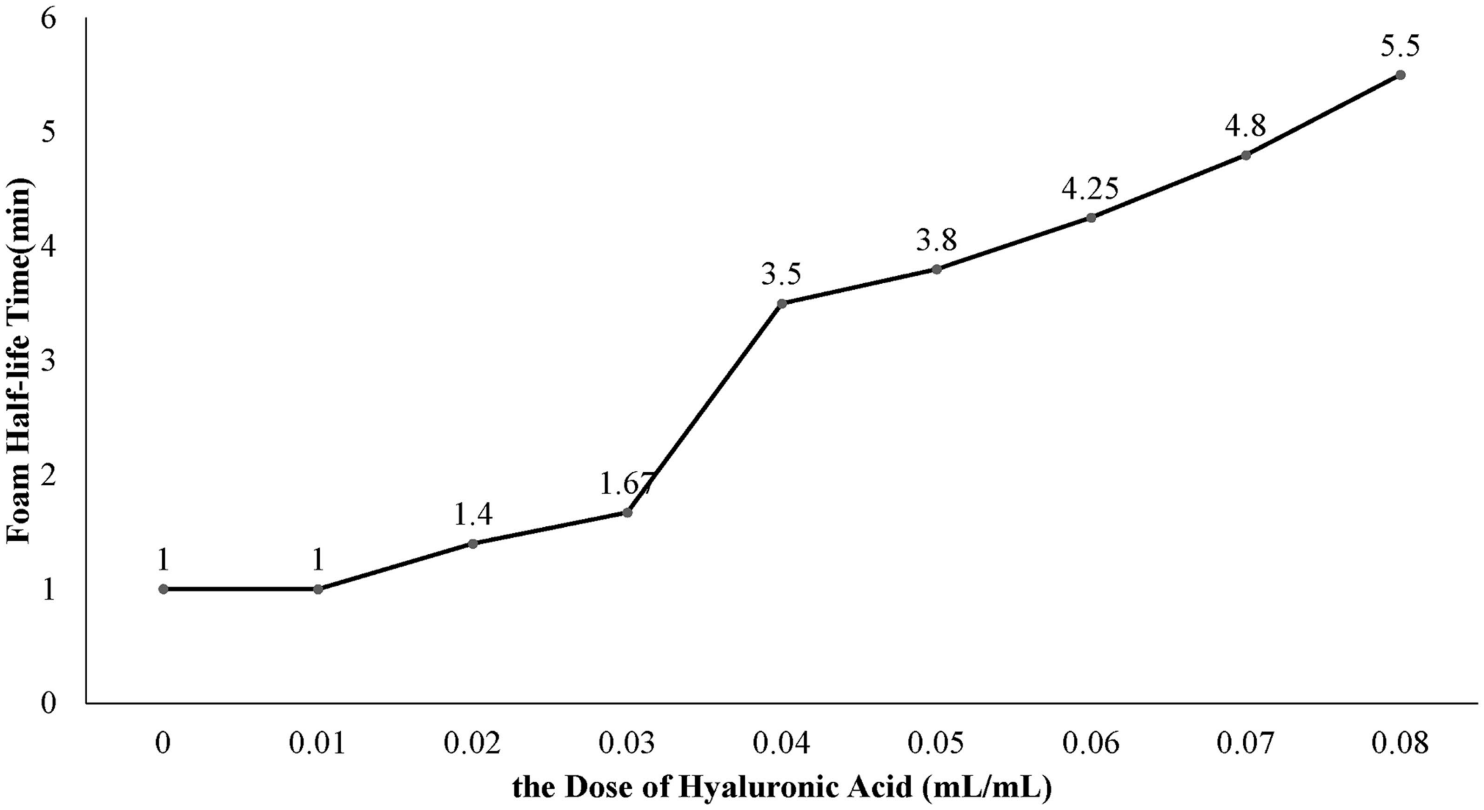
Effect of hyaluronic acid at different concentrations on ethanol foam half-life time.

We successfully developed an ethanol foam that is stable enough for foam sclerotherapy. When the fractional volume of ethanol reached 50% or higher, the solution could not be converted into a foam. When the ethanol concentration was lower than 50%, the foamability was dependent on the concentration of both ethanol and the surfactants. In experiment A, the foamability decreased as the concentration of ethanol increased and increased as the concentration of Tween 80 increased. In all the groups from experiment B, only a portion of the liquid in the syringe could form foam. In experiment C, all groups with ethanol concentrations lower than 50% produced foam. In experiment D, the FHT of the ethanol foam increased as the HA concentration increased (Figure 3). When the dose of HA was greater than 0.04 mL/mL, the foam was dense and homogeneous, with an FHT longer than 3.5 min. Hence, we considered an HA concentration of 0.04 mL/mL to be optimal for ethanol production.

## 4 Discussion

In this study, we have described a protocol to produce ethanol foam, a novel type of sclerosant. We were able to produce stable foam at an ethanol concentration of 48%. This could eliminate the need to use absolute ethanol for sclerotherapy and help avoid its serious side effects. The potential clinical application of this foam warrants investigation in future studies.

Our findings indicated that when the ethanol concentration exceeded 30%, the ethanol solution had a comparable effect on HUVECs to that of absolute ethanol. Thus, the optimal concentration of ethanol foam is higher than 30%. Additionally, we observed that solutions with ethanol concentrations below 60% had a minor impact on blood clotting. Hence, we surmised that ethanol foam with an ethanol concentration under 60% would not cause severe clotted blood and would produce fewer side effects than absolute ethanol. Based on these findings, we chose solutions with ethanol concentrations between 30 and 60% for foam production. Interestingly, we discovered that ethanol at concentrations less than 50% could be transformed into foam. It has been previously reported that ethanol molecules begin to aggregate at concentrations above 50% on account of changes in the microstructure of ethanol solutions that occur with changes in the concentration of ethanol (12, 13). Further, ethanol foam at concentrations over 48% was found to have poor stability. Considering the differences between *in vivo* and *in vitro* conditions, we determined that, among the experimental concentrations, a 48% fractional volume of ethanol was the ideal concentration at which the solution could be made into a stable foam and still have a potent effect.

A key factor affecting foam stability is the surfactant effect, which can reduce the surface tension and increase foaming efficiency (14). Therefore, we chose Tween 80, a type of surfactant that has good biocompatibility for use as a pharmaceutical adjuvant, according to the US Pharmacopoeia (15), to produce ethanol foam. To evaluate the foaming efficiency of Tween 80, the critical micelle concentration (CMC), a characteristic value that represents the formation of surfactant micelles, was adopted in our study. When the foam concentration is below the CMC, the foaming efficiency generally increases with increasing concentration until the concentration reaches or exceeds CMC slightly (14). In this study, we used Tween 80 at a concentration of 5 × 10^−3^ g/mL.

Mixtures of two or more surfactants have been demonstrated to have synergistic effects on micelle formation (14, 16), so we chose PC 80 as another surfactant to be combined with Tween 80. PC 80 is a pharmaceutical adjuvant approved by the FDA that is mainly used in pharmaceutical products as a dispersing, emulsifying, and stabilizing agent and is included in intravenous injections (15). Based on the findings in experiment B that there were no significant differences in foam formation between different concentrations of PC 80, we chose a lower concentration of 10^−2^ g/mL to prevent a decrease in the ethanol concentration.

It is recognized that high surface viscosity contributes to stable foam (17). Solutions with higher concentrations of Tween-80 have higher surface viscosity. However, excessive Tween 80 has also been associated with severe adverse effects (15), and it was recommended that its daily intake be restricted to 25 mg/kg body weight by the World Health Organization in 1974 (18). Considering this dose and the clinical use of Tween 80, we used a low concentration of Tween 80 (5 × 10^−3^ g/mL) to ensure safety. However, the ethanol foams prepared with 5 × 10^−3^ g/mL Tween 80 and 10^−2^ g/mL PC-80 exhibited poor stability. To enhance the stability of the foam, we added HA to the solution. HA, a type of biodegradable viscose that is nontoxic and nonimmunogenic (19), is reported to produce no generalized complications when intravenously injected as one of the elements of a foam sclerosant (20). The addition of HA was beneficial in our experiment, as the FHT increased with increasing concentrations of HA (Figure 3). We found that an HA concentration of 0.04 mL/mL is optimal for produce ethanol foam.

To conclude, we have developed a novel ethanol foam that has potential as a sclerosant for treating VMs. In the future, the positive response and good tolerance of this ethanol foam for treating VMs need to be proved in both animal experiments and clinical observation.

## Data availability statement

The original contributions presented in the study are included in the article/Supplementary material, further inquiries can be directed to the corresponding author.

## Ethics statement

The studies involving humans were approved by Research Ethics Committee of Qilu Hospital Shandong University. The studies were conducted in accordance with the local legislation and institutional requirements. The participants provided their written informed consent to participate in this study.

## Author contributions

H-SZ: Conceptualization, Formal analysis, Investigation, Methodology, Writing – original draft. Y-RL: Conceptualization, Formal analysis, Investigation, Methodology, Writing – original draft. S-HL: Conceptualization, Funding acquisition, Writing – review & editing.
