# Supplementary material for: Ethanol foam: a novel type of foam sclerosant for treating venous malformations
Source: Front Neurol. 2024 Aug 7;15:1431723. doi: 10.3389/fneur.2024.1431723 (PMC11339417; doi:10.3389/fneur.2024.1431723)
Supplement: Supplementary file 1 [file Table_1.DOCX]

| **n** | **Blank** | **Normal saline** | **10% ethanol** | **20% ethanol** | **30% ethanol** | **40% ethanol** | **50% ethanol** | **60% ethanol** | **70% ethanol** | **80% ethanol** | **90% ethanol** | **Absolute ethanol** |
| --- | --- | --- | --- | --- | --- | --- | --- | --- | --- | --- | --- | --- |
| 1 | 0.232 | 1.305 | 1.127 | 0.533 | 0.251 | 0.253 | 0.265 | 0.252 | 0.231 | 0.223 | 0.222 | 0.199 |
| 2 | 0.233 | 1.744 | 1.514 | 0.952 | 0.293 | 0.253 | 0.263 | 0.248 | 0.23 | 0.231 | 0.219 | 0.21 |
| 3 | 0.252 | 1.712 | 1.645 | 0.991 | 0.284 | 0.254 | 0.232 | 0.226 | 0.299 | 0.239 | 0.221 | 0.209 |
| 4 | 0.21 | 1.554 | 1.203 | 0.734 | 0.202 | 0.211 | 0.221 | 0.278 | 0.261 | 0.257 | 0.256 | 0.231 |
| 5 | 0.199 | 1.429 | 1.111 | 0.661 | 0.21 | 0.223 | 0.224 | 0.274 | 0.274 | 0.256 | 0.243 | 0.229 |
| 6 | 0.22 | 1.55 | 1.09 | 0.832 | 0.213 | 0.212 | 0.201 | 0.247 | 0.27 | 0.272 | 0.241 | 0.237 |

**Supplementary Table 1.** **The data of proliferation assay.**

The OD values of proliferation assay were obtained at 450 nm.
